# The oldest fossil record of Pseudopsinae from the Lower Cretaceous Yixian Formation of northeastern China (Coleoptera: Staphylinidae: Pseudopsinae)

**DOI:** 10.1038/s41598-022-08450-3

**Published:** 2022-03-17

**Authors:** Yuchu Liu, Erik Tihelka, Chenyang Cai, Li Tian

**Affiliations:** 1 State Key Laboratory of Biogeology and Environmental Geology, China University of Geosciences, Wuhan, 430078 China; 2 School of Earth Science, University of Bristol, Bristol, BS8 1TQ UK; 3 State Key Laboratory of Palaeobiology and Stratigraphy, Nanjing Institute of Geology and Palaeontology, and Center for Excellence in Life and Paleoenvironment, Chinese Academy of Sciences, Nanjing, 210008 China

**Keywords:** Palaeontology, Entomology

## Abstract

The Cretaceous witnessed a radiation of rove beetles (Staphylinidae), the most species-rich beetle family. Although most staphylinid subfamilies have been documented from Cretaceous strata over the world, there has been no fossil record of the subfamily Pseudopsinae until a recently reported fossil from the 99-Ma-old Myanmar amber. Here we describe a new compression fossil from the Lower Cretaceous Yixian Formation of northeastern China. It is described as *Cretaceonanobius fossilis.* gen. et sp. nov. and assigned to the extant subfamily Pseudopsinae, based on the well-preserved carinae on the pronotum, a carina on ventrites II and III, and distinctly separated mesocoxae. The discovery of *Cretaceonanobius fossilis* gen. et sp. nov. backdates the earliest fossils record of Pseudopsinae to 125 Ma in the Northern Hemisphere and sheds new lights on the evolution history and paleobiogeography of this subfamily.

The rove beetles, Staphylinidae, are not only one of the most abundant beetle families, but also the most speciose family in the animal kingdom, with over 65,000 described species in 33 subfamilies^1,2^. The oldest fossil record of Staphylinidae dates back to the Middle Jurassic and are represented by exceptional fossils known from the Haifanggou Formation in northeastern China (~ 165 Ma)^3–5^. During the Jurassic, taxa belonging to seven subfamilies have been reported, primarily from low and middle latitudes of the Northern Hemisphere^6–10^, as well as Australia^10^. While molecular clock studies support a Jurassic radiation of rove beetles^12^, it is not until the Cretaceous that Staphylinidae become diverse in the fossil record, especially thanks to well-preserved specimens from exceptional deposits in east Asia such as the amber from northern Myanmar and shales of the Yixian Formation in China^13,14^, along other outcrops worldwide that await more detailed study. Currently, seven Staphylinidae subfamilies trace their earliest appearance in the fossil record to Myanmar amber^8,15–19^. Five rove beetle subfamilies, namely Piestinae, Oxyporinae, Paederinae, Staphylininae, and Tachyporinae as well as species of uncertain subfamilial attribution, have been reported from the Yixian Formation, represented by 34 described species in total^10,20–24^.

Pseudopsinae is a comparatively small and moderately diverse staphylinid subfamily belonging to the Staphylinine group of subfamilies^25^. It is represented in the Recent fauna by four genera with 55 species. *Pseudopsis* Newman is by far the largest genus comprising 51 species distributed in the Holarctic, Neotropical, and north parts of the Oriental regions as well as on New Zealand. The remaining genera, *Zalobius* LeConte, *Asemobius* Horn, and *Nanobius* Herman are restricted to the western Nearctic Region^26^. Up until now, the only unequivocal fossil pseudopsine beetle has been *Cretopseudopsis maweii* Liu, Tihelka, Tian, Huang & Cai described from the Cretaceous (*ca*. 99 Ma) amber from norther Myanmar^19^. Here we describe a new compression fossil from the Yixian Formation, that further pushes back the oldest fossil record of Pseudopsinae to the Early Cretaceous.

## Results

### Systematic palaeontology

Order Coleoptera Linnaeus, 1758

Family Staphylinidae Latreille, 1802

Subfamily Pseudopsinae Ganglbauer, 1895

**Genus *Cretaceonanobius*** Liu, Tihelka, Cai et Tian, gen. nov.

*Type species. Cretaceonanobius fossilis* sp. nov.

*Diagnosis*. Body medium sized. Head small, eyes large. Antennae short, located on the front of the head. Distinctly constricted neck region present. Pronotum suborbicular, with a smooth margin, longitudinal carinae of the pronotum, widest in anterior half. Elytra short, together slightly longer than wide, covering only part of tergite II, exposing rest of abdomen.

*Etymology*. The generic name is a combination of the Latin ‘*Cretaceo-*’, after the age of the fossil, and the genus *Nanobius*.

***Cretaceonanobius fossilis*** Liu, Tihelka, Cai et Tian, sp. nov.

(Figs. 2, 3)

*Type material*. Holotype, NIGP177043a, b. Part and counterpart with dorsal and ventral structures visible.

**Figure 1:**
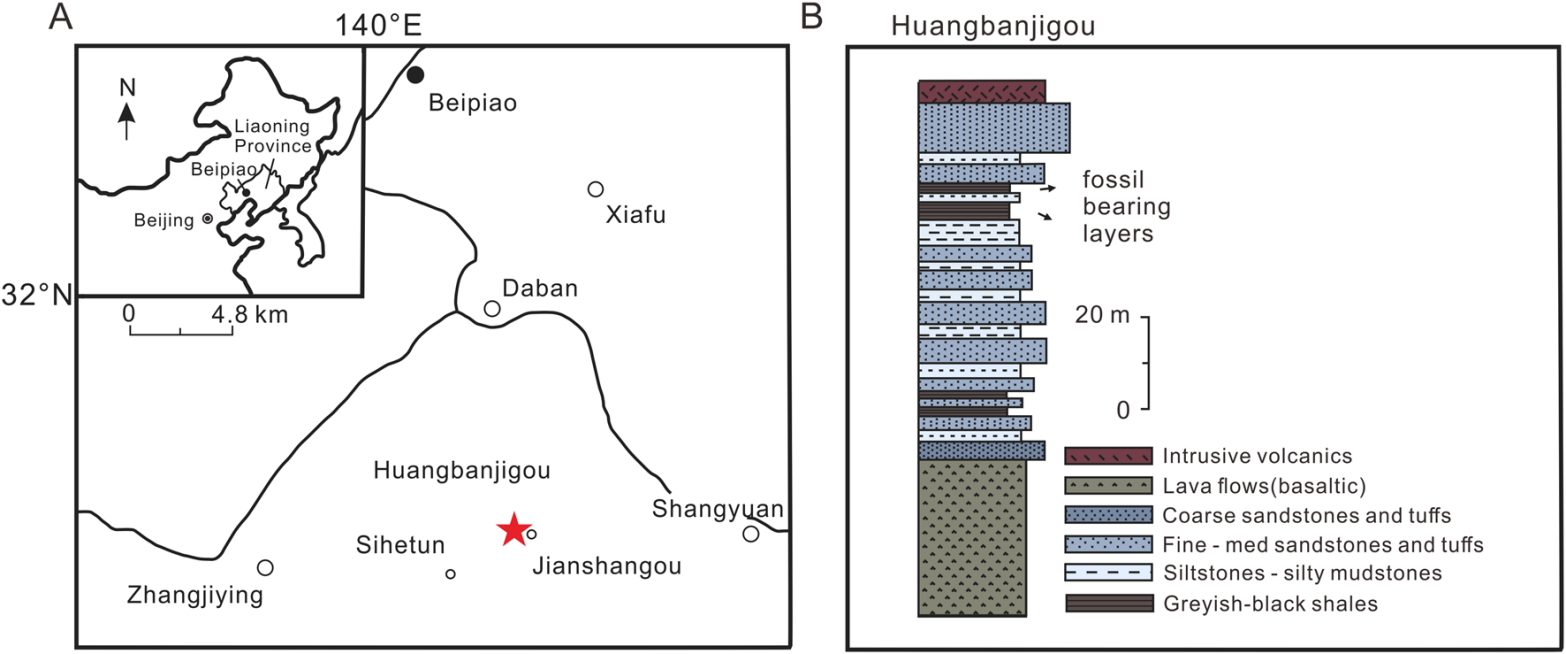
Geographical and stratigraphic context of the Yixian Formation, after Liu et al.^54^. (**A**) Map of the western Liaoning Province, ^56^ with the red star indicating the fossil locality at Huangbanjigou (modified from Cai et al.^55^). (**B**) Fossiliferous strata exposed in Huangbanjigou (modified from Wang et al.^56^).

*Locality and horizon*. Huangbanjigou, Beipiao City of Liaoning Province, northeast China; Lower Cretaceous Yixian Formation (Fig. 1A,B).

*Etymology*. The specific epithet refers to the fossil nature of the taxon.

*Diagnosis*. As for the genus (vide supra) and a combination of coarse punctuation of the head and pronotum, pronotum with longitudinal carinae.

**Figure 2:**
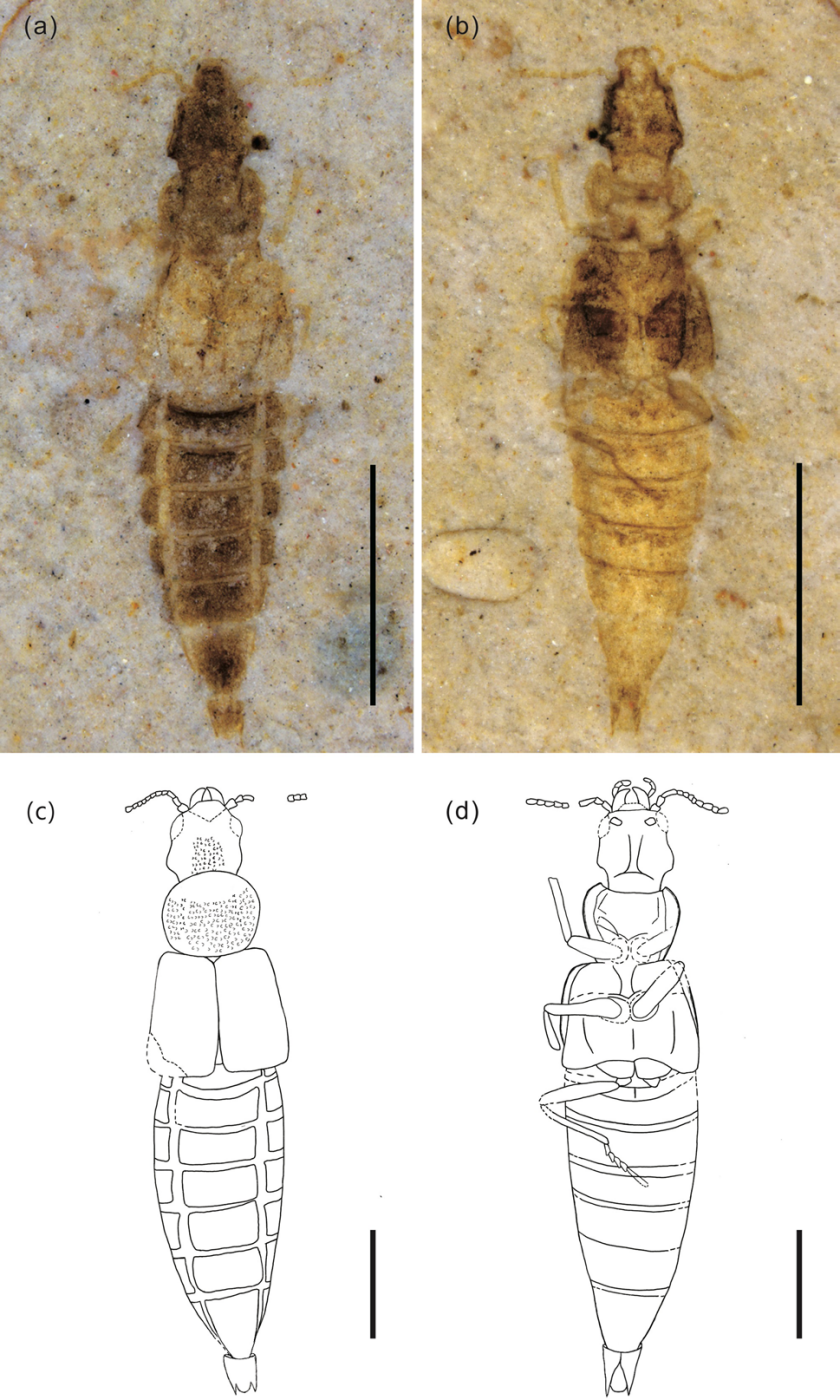
Habitus of *Cretaceonanobius fossilis* gen et sp. nov. (holotype, NIGP 177043), part (**a**) (dorsal) and counterpart (**b**) (ventral) moistened with 75% alcohol under low angle lighting; (**c**) body from dorsal side line in drawing and (**d**) body from ventral side in drawing. Scale bars = 2 mm in (**a**), (**b**) and 1 mm in (**c**), (**d**).

*Description*. Body medium sized and elongate, 5.75 mm long from clypeus to abdominal apex (Fig. 2).

**Figure 3:**
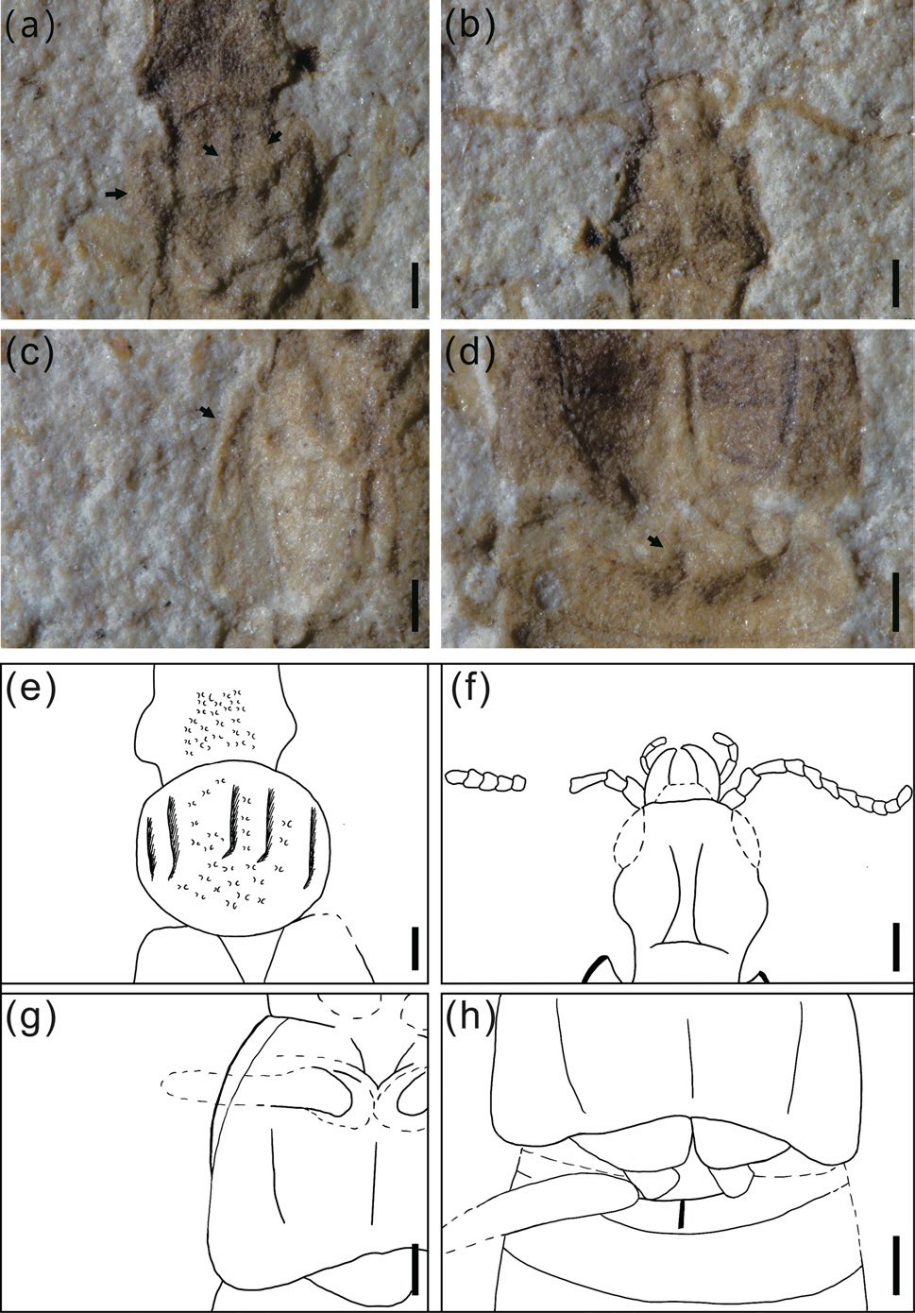
Morphological details of *Cretaceonanobius fossilis* gen. et sp. nov. (holotype, NIGP 177043). (**a**) pronotum of the part (arrows, carina on the pronotum); (**b**) head, ventral; (**c**) left elytron (arrow, hypomeron); (**d**) base of abdomen (arrow, intermetacoxal carina); (**e**) same as (**a**), line drawing; (**f**) same as (**b**), line drawing; (**g**) same as (**c**), line drawing; (**h**) same as (**d**), line drawing. Scale bars = 200 μm.

**Figure 4:**
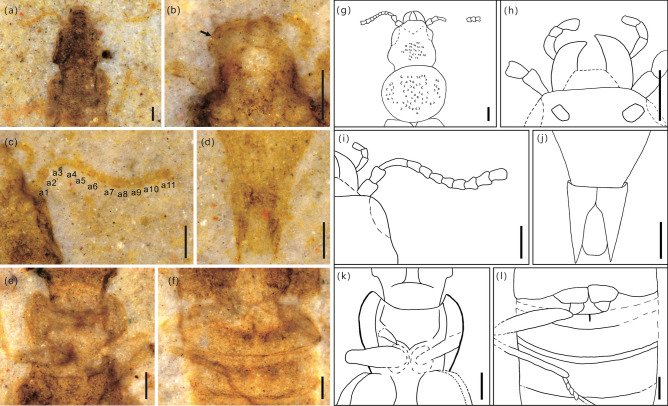
Morphological details of *Cretaceonanobius fossilis* gen. et sp. nov. (holotype, NIGP 177043). (**a**) Head, dorsal (arrow, punctation on head); (**b**) head, ventral (arrow, maxillary palp); (**c**) antenna; (**d**) latero-apical parts of segment X apex, ventral; (**e**) neck and procoxae; (**f**) metacoxae and metathoracic leg; (**g**) same as (**a**), line drawing; (**h**) same as (**b**), line drawing; (**i**) asame as (**c**), line drawing; (**j**) same as (**d**), line drawing; (**k**) same as (**e**), line drawing; (**l**) same as (**f**), line drawing. Abbreviations: a = antennomere; m = mandible; prc = procoxa. Scale bars = 200 μm.

Head, including mandibles, 0.97 mm long, broadest at eyes (Fig. 4a,g). Mandibles robust, curved mesally (Figs. 3b,f, 4b,h). Maxillary palp elongate, 4-segmented (Fig. 2b,d). Antennae filiform and narrow, 0.78 mm long, 11-segmented, without club. Antennomeres 1–3 elongate, about 1.2 times longer than wide, (Fig. 4c,i). All antennomeres symmetrical. Dorsum of head densely punctate (Fig. 2a,c). Compound eyes moderate in size. Ocelli absent. Gular sutures visible. Neck narrow, constricted (Fig. 4e,k).

Pronotum 0.73 mm long, 1.28 times as long as wide, about 0.52 times as long as elytra. Anterior pronotal angles rounded. Pronotum approximately suborbicular, widening anteriorly, with five carinae distributing on it (Fig. 3a,e). Posterior pronotal angles rounded. Prosternum short, procoxae contiguous, suborbicular (Fig. 2b,d). Mesocoxae ovate, contiguous, suborbicular. Protrochantin concealed. Mesoventral posterior and metaventral anterior processes present. Metacoxae narrowly separated.

Elytra short, 1.38 mm long, 1.14 times wider than long. Elytral surface coarse punctation and longitudinal carinae, hypomeron visible in ventral view (Fig. 3c,g).

Legs long, slender (Fig. 4f,l). Abdomen 3.65 mm long, with six visible sternites. Six tergites visible dorsally. Intercoxal process of sternite II triangular (Fig. 3d,h). Tergites III–VII subequal in length and each with one pair of laterosternites, segments VII and VIII gradually narrowed, tergite VIII subtriangular, tergite X with large rearward extension (Fig. 4d,j). Abdomen with rows of subtriangular sculptures. Two tail spines present.

## Discussion

### Systematic placement

The new fossil can be excluded from all extant staphylinid families, with the exception of Pseudopsinae. Its elongate body and antennae located on the front of the head, differentiate the new taxon, from the known members of Mesozoic Staphylininae and Aleocharinae^27,28^. Based on the lack of a pair of ocelli and a narrower body shape, the fossil is excluded from Omaliinae^29^. The lack of large eyes and distinctly clubbed antennae, distinguish *Cretaceonanobius* gen. nov. from Megalopsidiinae and Steninae^30,31^. Clubbed antennae, enlarged maxillary palpomere 3, and 3-segmented tarsi separate Protopselaphinae, to which *Cretaceonanobius fossilis* gen. et sp. nov. cannot be assigned^27^. Robust mandibles and a distinct labial palpus differentiate Oxyporinae and Euaesthetinae from *Cretaceonanobius fossilis* gen. et sp. nov.^29,32^. The pronotum of the *Cretaceonanobius fossilis* gen. et sp. nov. is suborbicular and widest medially, showing significant differences with the known Mesozoic members of Trigonurinae and Tachyporinae, which are characterized by the pronotum broadest at the base^29,33^. Compared with Proteininae, the *Cretaceonanobius fossilis* gen. et sp. nov. has a narrower body, with six tergites exposed^29,30^. The antennae without a distinct apical club or dense setation shows it is not a member of Trichophyinae or Habrocerinae^34,35^. The antennae of Olisthaerinae are located on the lateral side of the forehead^29^. Different from the suborbicular pronotum of *Cretaceonanobius fossilis* gen. et sp. nov., the pronotum on Piestinae is invertedly trapezoidal and the head large^27^. Based on the lack of paratergites in Osoriinae and two pairs of paratergites in Olisthaerinae, *Cretaceonanobius fossilis* gen. et sp. nov. with only a pair of paratergites can be distinguished from both subfamilies^27,29,36,37^. Habrocerinae are distinguished from the present fossil by asymmetrical mandibles Meanwhile, the extant subfamily Habrocerinae, which antennae 3–11 are slender and covered with bristles, is different from the *Cretaceonanobius fossilis* gen. et sp. nov.

*Cretaceonanobius fossilis* gen. et sp. nov. possesses derived characters such as the punctation on the temple and the spines on the pronotum which unite it with Pseudopsinae to the exclusion of the superficially similar Solieriinae. Based on this combination of characters, we assign *C. fossilis* gen. et sp. nov. to Pseudopsinae.

The general habitus of *Cretaceonanobius* gen. nov. resembles *Pseudopsis* and *Nanobius*^36,38,39^. Like many species of the genus *Pseudopsis*, the fossil has a single pair of laterosternites (although in *Nanobius* the second outside pair is very narrow and easily overlooked). Similar to *Nanobius*, it has a very distinct neck, pronotum with distinctly carinae, dense deep punctation on the head and pronotum and has the indication of a carina on sternites II-III. Unlike both genera, which have distributed carinae on the elytra, *Cretaceonanobius* gen. nov. lacks carinae on the elytra. Some modern *Pseudopsis* such as *Pseudopsis obliterata* LeConte, 1879 also lack carinae, although they have rounded elevations and uneven surfaces on the pronotum and elytra.

*Cretaceonanobius fossilis* gen. et sp. nov. is differentiated from *Asemobius*, which possesses a triangular head with long maxillary palps, neck concealed dorsally, and pronotum broadest in the anterior third. Furthermore, *Zalobius* is differentiated by its shape of the pronotum^39^. *C. fossilis* gen. et sp. nov. further differs from the Cretaceous *Cretopseudopsis maweii* from Myanmar amber in that the protrochantins of the latter are barely visible, elytra are carinate, and abdominal terga lack basolateral ridges^19^. Both genera however share a deeply punctate head surface, very distinct neck. Predating *Cretopseudopsis* from Myanmar amber, *Cretaceonanobius fossilis* gen. et sp. nov. represents the earliest fossil record of Pseudopsinae so far.

### Geography and habitat evolution history of Pseudopsine

**Figure 5:**
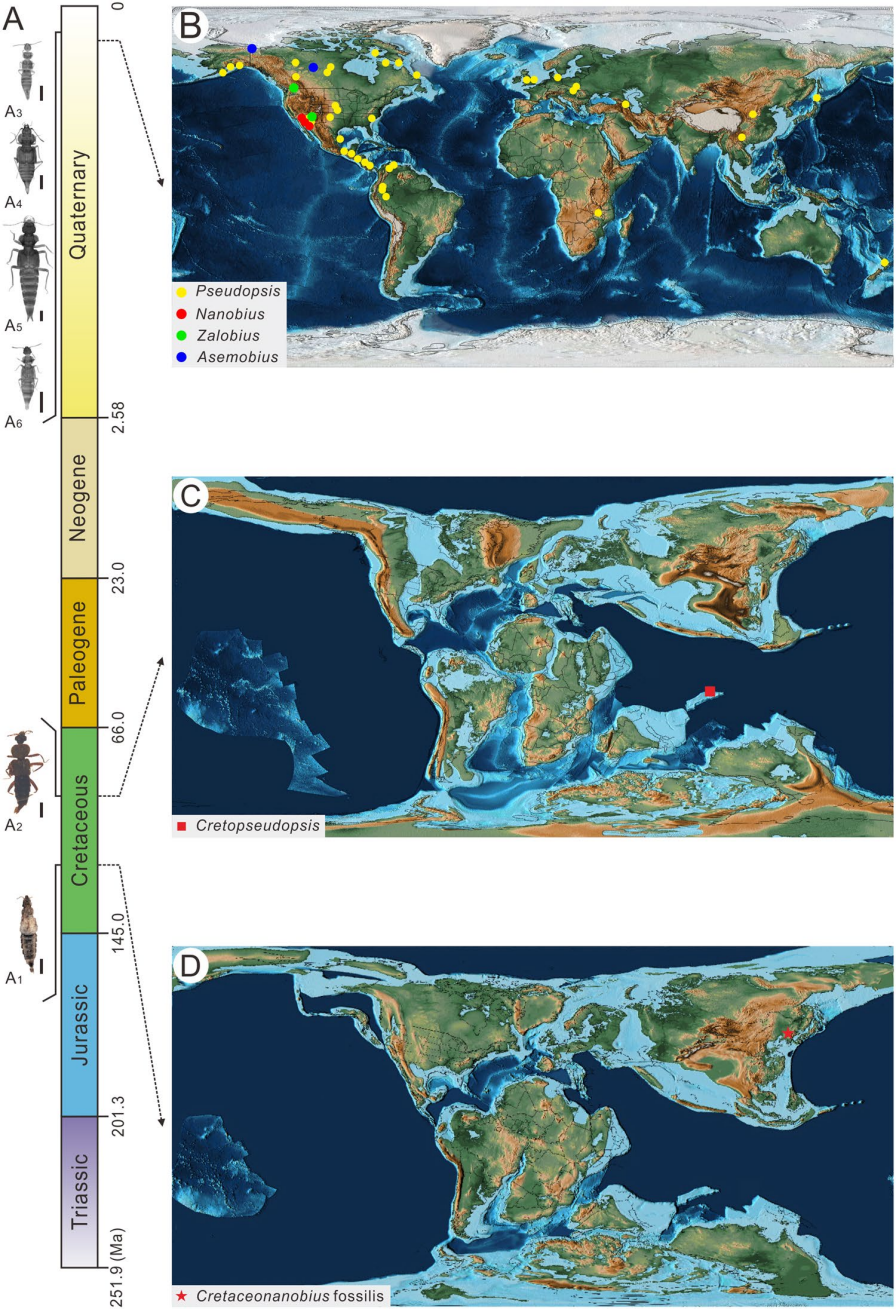
Distribution of extinct and extant Pseudopsinae taxa. (**A**_**1**_) *Cretopseudopsis maweii*, (**A**_**2**_) *Cretaceonanobius fossilis* gen. et sp. nov., (**A**_**3**_) *Pseudopsis*, (**A**_**4**_) *Asemobius*, (**A**_**5**_) *Nanobius*, (**A**_**6**_) *Zalobius*. Scale bars = 500 μm. (**B**) Recent geographic map after Scotese^57^: yellow, red, green and blue dots show the distribution of *Pseudopsis*, *Nanobius*, *Zalobius* and *Asemobius*, respectively; (**C**) Palaeogeographic map of the Early Cretaceous after Scotese^57^, with the red square for *Cretopseudopsis maweii*; (**D**) Palaeogeographic map of the Early Cretaceous after Scotese^57^, with the red star indicating the distribution of the *Cretaceonanobius fossilis* gen. et sp. nov.

Extant Staphylinidae are widely distributed on all continents except Antarctica, and contribute a large share of animal biodiversity in microhabitats such as leaf litter and the soil^40^. While the rise of angiosperms during the Cretaceous, known as the Cretaceous Terrestrial Revolution or Angiosperm Terrestrial Revolution, has been linked with the diversification of some beetle lineages, it remains unclear to what extent it impacted the macroevolutionary dynamics of rove beetles^41,42^. Although a Triassic–Jurassic origin of crown-Staphylinidae supported by molecular clock studies is congruent with the fossil record^11^, in the timing of the origin of individual subfamilies is less certain^42^. As such, discoveries of rove beetles from the early Cretaceous and Jurassic deposits that predate the widespread appearance of flowering plants are important for calibrating the timescale of staphylinid evolution and testing the impacts of the Cretaceous Terrestrial Revolution on the diversification of beetles. The four Pseudopsine genera are widely distributed. *Zalobius* is known from southern British Columbia south to central California (Fig. 5, black circle). *Nanobius* and *Asemobius* occur from southern British Columbia to southern California and southwest California (Fig. 5, red dots; purple dots). *Pseudopsis* is distributed in the Nearctic, Palearctic, Neotropical, northern Oriental, and Australasian regions^43,44^. All four extant genera mainly live in middle and low latitudes and partly in high latitudes. All known Mesozoic Pseudopsinae from Myanmar amber and the Yixian Formation occurred in Equatorial and mid-latitude and regions, respectively^45,46^.

Extant pseudopsines inhabit leaf litter as well as flood debris and grass growing near streams, from coastal areas to mountainous regions^38,39^. The Myanmar amber palaeoenvironment has been reconstructed as a tropical forest standing at the seashor^47^ suggesting that *Cretopseudopsis maweii* lived in coastal environments (Fig. 5B). The spore fossils from the Yixian Formation overwhelmingly belong to gymnosperms, the dominant vegetation type was a humid coniferous forest^48^. The humid climatic conditions also reflected by the woodstone^48,49^. At the same time, the fossils of conchostraca were found in the Yixian Formation, which lived in shallow water and reflected a semi-arid to semi-humid climate^13^. The insects found in the Yixian Formation included terrestrial, aquatic and semi-aquatic species, which indicated that there presents land, still water and humid living conditions in the Beipiao at that time^50^. The feeding habits of Pseudopsinae shows the living environment of *Cretaceonanobius fossilis* gen. et sp. nov. may in the swampy area which surrounded by ancient lake basin and trees grew on the high mountains nearby, and it is consistent with the paleo-environment reflected by the fossil assemblage of the Yixian Formation^13^. The diverse palaeoenvironments occupied by fossil pseudopsines reflect their broad ecological niche during the Cretaceous.

## Material and methods

The studied specimen originates from the Yixian Formation at Huangbanjigou village, Beipiao City in the Liaoning Province of China^13^. The Yixian Formation is well known for the discoveries of numerous well-preserved dinosaurs, mammals, birds, angiosperms, and insect fossils^46,51,52^ (Jehol Biota). Its age has been constrained to be middle Early Cretaceous, circa 125 Ma, by ^40^Ar/^39^Ar dating^53^.

The specimen is compressed in shales, both ventral and dorsal sides have been well preserved (Fig. 1), including morphological details such as the punctation of the head and a single pair of paratergites. Photographs were taken with a Zeiss Discovery V20 microscope equipped with a digital camera after the specimen has been moistened with 75% ethanol. The type specimen is deposited in the Nanjing Institute of Geology and Palaeontology, Chinese Academy of Sciences, Nanjing, China. New nomenclatural acts established have been registered in ZooBank under the publication LSID urn:lsid: sid:zoobank.org:act:B4882FCA-1EF8-4A43-8A54-5867FB05C060.

## References

[1] Grebennikov VV, Newton AF (2009). Good-bye Scydmaenidae, or why the ant-like stone beetles should become megadiverse Staphylinidae sensu latissimo (Coleoptera). Eur. J. Entomol..

[2] Yamamoto S, Takahashi Y (2019). First and oldest Leptochirini rove beetles illuminate diverse cephalic structures in the Cretaceous (Coleoptera: Staphylinidae: Osoriinae). Syst. Entomol..

[3] Cai C (2022). Integrated phylogenomics and fossil data illuminate the evolution of beetles. R. Soc. Open Sci..

[4] Fikáček M (2020). Reliable placement of beetle fossils via phylogenetic analyses – Triassic *Leehermania* as a case study (Staphylinidae or Myxophaga?). Syst. Entomol..

[5] Cai C (2014). Early origin of parental care in Mesozoic carrion beetles. Proc. Natl. Acad. Sci..

[6] Ryvkin AB (1985). Beetles of the family Staphylinidae from the Jurassic of Transbaikalia. Trudy Paleontologicheskogo Instituta, Akademia nauk SSSR.

[7] Tikhomirova AL, Rohdendorf BB (1968). Staphylinid beetles of the Jurassic of the Karatau (Coleoptera, Staphylinidae). Jurassic Insects of the Karatau.

[8] Cai C, Huang D (2015). The oldest osoriine rove beetle from Cretaceous Burmese amber (Coleoptera: Staphylinidae). Cretaceous Res..

[9] Weyenbergh H (1869). Sur les insectes fossiles du calcaire lithographique de la Baviere, qui se trouvent au Musee Teyler. Extrait des Archives du Musee Teyler.

[10] Cai C-Y, Huang D-Y (2014). Diverse oxyporine rove beetles from the Early Cretaceous of China (Coleoptera: Staphylinidae). Syst. Entomol..

[11] Cai C-Y, Yan EV, Beattie R, Wang B, Huang D-Y (2013). First rove beetles from the Jurassic Talbragar Fish Bed of Australia (Coleoptera, Staphylinidae). J. Paleontol..

[12] Lü L (2020). Linking evolutionary mode to palaeoclimate change reveals rapid radiations of staphylinoid beetles in low-energy conditions. Curr. Zool..

[13] Jiang B, Sha J (2007). Preliminary analysis of the depositional environments of the Lower Cretaceous Yixian Formation in the Sihetun area, western Liaoning, China. Cretaceous Res..

[14] Cruickshank RD, Ko K (2003). Geology of an amber locality in the Hukawng Valley, Northern Myanmar. J. Asian Earth Sci..

[15] Clarke DJ, Chatzimanolis S (2009). Antiquity and long-term morphological stasis in a group of rove beetles (Coleoptera: Staphylinidae): Description of the oldest Octavius species from Cretaceous Burmese amber and a review of the “Euaesthetine subgroup” fossil record. Cretaceous Res..

[16] Cai C-Y, Huang D-Y (2014). The oldest micropepline beetle from Cretaceous Burmese amber and its phylogenetic implications (Coleoptera: Staphylinidae). Naturwissenschaften.

[17] Chatzimanolis S, Engel MS, Newton AF, Grimaldi DA (2010). New ant-like stone beetles in mid-Cretaceous amber from Myanmar (Coleoptera: Staphylinidae: Scydmaeninae). Cretaceous Res..

[18] Thayer MK, Newton AF, Chatzimanolis S (2012). *Prosolierius*, a new mid-Cretaceous genus of Solieriinae (Coleoptera: Staphylinidae) with three new species from Burmese amber. Cretaceous Res..

[19] Liu Y, Tihelka E, Tian L, Huang D, Cai C (2020). First fossil pseudopsine rove beetle from mid-Cretaceous Burmese amber (Coleoptera: Staphylinidae: Pseudopsinae). Zootaxa.

[20] Yue Y, Ren D, Solodovnikov A (2011). The oldest fossil species of the rove beetle subfamily Oxyporinae (Coleoptera: Staphylinidae) from the Early Cretaceous (Yixian Formation, China) and its phylogenetic significance. J. Syst. Palaeontol..

[21] Yue YL, Ren D, Solodovnikov A (2010). *Megolisthaerus chinensis* gen. et sp. n (Coleoptera: Staphylinidae incertae sedis): An enigmatic rove beetle lineage from the Early Cretaceous. Insect Syst. Evol..

[22] Yue Y, Makranczy G, Ren D (2012). A Mesozoic species of *Anotylus* (Coleoptera, Staphylinidae, Oxytelinae) from Liaoning, China, with the earliest evidence of sexual dimorphism in rove beetles. J. Paleontol..

[23] Solodovnikov A, Yue Y, Tarasov S, Ren D (2013). Extinct and extant rove beetles meet in the matrix: Early Cretaceous fossils shed light on the evolution of a hyperdiverse insect lineage (Coleoptera: Staphylinidae: Staphylininae). Cladistics.

[24] Yue Y, Gu J-J, Yang Q, Wang J, Ren D (2016). The first fossil species of subfamily Piestinae (Coleoptera: Staphylinidae) from the Lower Cretaceous of China. Cretaceous Res..

[25] Thayer MK, Beutel RG, Leschen RAB (2016). Staphylinidae. Handbook of Zoology. Vol. IV. Part 38. 2nd Edition. Coleoptera. Vol. 1. Morphology and Systematics (Archostemata, Adephaga, Myxophaga, Staphyliniformia, Scarabaeiformia, Elateriformia).

[26] Newton AF, Thayer MK (2003). Catalog of Higher Taxa of Staphyliniformia and Genera and Subgenera of Staphylinoidea.

[27] Thayer, M. Staphylinoidea. [chapter] 11.7. Staphylinidae Latreille, 1802. *Morphol. Syst. Archostemata Adephaga Myxophaga Polyphaga Partim* 296–344 (2005).

[28] Ashe JS (2005). Phylogeny of the tachyporine group subfamilies and ‘basal’ lineages of the Aleocharinae (Coleoptera: Staphylinidae) based on larval and adult characteristics: Phylogeny of tachyporine group staphylinids. Syst. Entomol..

[29] Newton AF, Thayer MK, Ashe JS, Chandler DS, Arnett RH, Thomas MC (2001). Staphylinidae Latreille, 1802. American Beetles.

[30] Newton AF, Thayer MK, Pakaluk J, Slipinski SA (1995). Protopselaphinae new subfamily for *Protopselaphus* new genus from Malaysia, with a phylogenetic analysis and review of the Omaliine Group of Staphylinidae including Pselaphidae. Biology, Phylogeny, and Classification of Coleoptera: Papers Celebrating the 80th Birthday of Roy A.

[31] Eichelbaum F (1915). Verbesserungen und Zusatze zu meinem Katalog der Staphylinidengattungen aus dem Jahre 1909. Archiv für Natur. (A).

[32] Hanley RS, Goodrich MA (1995). Review of Mycophagy, Host Relationships and Behavior in the New World Oxyporinae (Coleoptera: Staphylinidae). Coleopt. Bull..

[33] Herman LH (2001). Catalog of the Staphylinidae (Insecta: Coleoptera). 1758 to the End of the Second Millennium. VI. Staphylinine Group (Part 3) Staphylininae: Staphylinini (Quediina, Staphylinina, Tanygnathinina, Xanthopygina), XantholininiStaphylinidae Incertae SedisFossils, Protactinae†. Bull. Am. Mus. Nat. Hist..

[34] Ashe JS, Newton AF (1993). Larvae of *Trichophya* and phylogeny of the Tachyporine Group of subfamilies (Coleoptera: Staphylinidae) with a review, new species and characterization of the Trichophyinae. System. Ent..

[35] Muir F (1919). The male abdominal segments andaedeagus of *Habrocerus capillaricornis* Grav (Coleoptera, Staphylinidae). Trans. Ent. Soc. Lond..

[36] Newton AF (1982). Redefinition, revised phylogeny, and relationships of Pseudopsinae (Coleoptera, Staphylinidae). Am. Mus. Novit..

[37] Moore I, Legner EF (1973). The genera of the subfamilies Phloeocharinae and Olisthaerinae of America north of Mexico with description of a new genus and new species from Washington (Coleoptera: Staphylinidae). Can. Entomol..

[38] Herman LH (1975). Revision and phylogeny of the monogeneric subfamily Pseudopsinae for the world (Staphylinidae, Coleoptera). Bull. Am. Mus. Nat. Hist..

[39] Herman LH (1977). Revision and phylogeny of *Zalobius*, *Asemobius*, and *Nanobius*, new genus (Coleoptera, Staphylinidae, Piestinae). Bull. Am. Mus. Nat. Hist..

[40] Parker J (2017). Staphylinids. Curr. Biol..

[41] Lloyd GT (2008). Dinosaurs and the cretaceous terrestrial revolution. Proc. R. Soc. B Biol. Sci..

[42] Benton MJ, Wilf P, Sauquet H (2021). The Angiosperm Terrestrial Revolution and the origins of modern biodiversity. New Phytol..

[43] Newton AF, Roskov Y (2020). StaphBase: Staphyliniformia world catalog database (version, Nov 2018). Species 2000 & ITIS Catalogue of Life.

[44] Yin Z-W (2021). Two new species and additional records of *Pseudopsis* Newman from China (Coleoptera: Staphylinidae: Pseudopsinae). J. Nat. Hist..

[45] Westerweel J (2019). Burma Terrane part of the Trans-Tethyan arc during collision with India according to palaeomagnetic data. Nat. Geosci..

[46] Ren D, Shih C, Gao T, Wang Y, Yao Y (2019). Rhythms of Insect Evolution: Evidence from the Jurassic and Cretaceous in Northern China.

[47] Grimaldi D, Ross AJ, Fraser NC, Sues H-D (2017). Extraordinary Lagerstätten in amber, with particular reference to the Cretaceous of Burma. Terrestrial Conservation Lagerstätten: Windows into the Evolution of Life on Land.

[48] Yu, J.X., Pu, R.G., Wu, H.Z., Sporo-pollen assemblages from the upper part of the Rehe group, Liaoning Province. *Acta Geo. Sin.* **2** (1989).

[49] Zheng SL, Zheng YJ, Xing DH (2003). Characteristics age and climate of Late Jurassic Yixian Flora from Western Liaoning. J. Stratigr..

[50] Zhang H, Wang B, Fang Y (2010). Evolution of insect diversity in the Jehol Biota. Sci. China Earth Sci..

[51] Pan Y, Sha J, Zhou Z, Fürsich FT (2013). The Jehol Biota: Definition and distribution of exceptionally preserved relicts of a continental Early Cretaceous ecosystem. Cretaceous. Res..

[52] Xu X, Zhou Z, Wang Y, Wang M (2020). Study on the Jehol Biota: Recent advances and future prospects. Sci. China Earth Sci..

[53] Swisher CC, Wang YQ, Wang XL, Xu X, Wang Y (1999). Cretaceous age for the feathered dinosaurs of Liaoning, China. Nature.

[54] Liu Y (2021). A transitional fossil sheds light on the early evolution of the Staphylinine group of rove beetles (Coleoptera: Staphylinidae). J. Syst. Palaeontol..

[55] Cai C, Huang D, Solodovnikov A (2011). A new species of *Hesterniasca* (Coleoptera: Staphylinidae: Tachyporinae) from the Early Cretaceous of China with discussion of its systematic position. Insect Syst. Evol..

[56] Wang X, Wang Y, Zhang F, Zhang J, Zhou Z, Jin F, Hu Y, Gu G, Zhang H (2000). Vertebrate biostratigraphy of the Lower Cretaceous Yixian formation in Lingyuan, western Liaoning and its neighboring southern Nei Mongol (inner Mongolia). China. Vert. PalAs..

[57] Scotese, C.R., Dreher, C. *GlobalGeology* (2012). http:/www.GlobalGeology.com.

